# Anti-Atopic Activities of *Sargassum horneri* Hot Water Extracts in 2,4-Dinitrochlorobezene-Induced Mouse Models

**DOI:** 10.4014/jmb.2211.11007

**Published:** 2023-01-13

**Authors:** Ga-Eun Woo, Hye-Ji Hwang, A-Yeoung Park, Ji-Yoon Sim, Seon-Young Woo, Min-Ji Kim, So-Mi Jeong, Nak-Yun Sung, Dong-Sub Kim, Dong-Hyun Ahn

**Affiliations:** 1 Department of Food Science and Technology/Institute of Food Science, Pukyong National University, Busan 48513, Republic of Korea; 2 Institute of Fisheries Sciences, Pukyong National University, Busan 48513, Republic of Korea; 3 Division of Natural Product Research, Korea Prime Pharmacy CO., LTD., Jeonnam 58144, Korea

**Keywords:** Th1, Th2, atopic dermatitis, *Sargassum horneri*, skin severity score, IgE

## Abstract

Atopic dermatitis (AD) is a chronic inflammation associated with skin hypersensitivity caused by environmental factors. The objent of this study was to assess the hot water extracts of *Sargassum horneri* (SHHWE) on AD. AD was induced by spreading 2,4-dinitrochlorobenzene (DNCB) on the BALB/c mice. The efficacy of SHHWE was tested by observing the immunoglobulin E (IgE), cytokine, skin clinical severity score and cytokine secretions in concanavalin A (Con A)-stimulated splenocytes. The levels of interleukine (IL)-4, IL-5 and IgE, the pro-inflammatory cytokines that are closely related, were notably suppressed in a does-dependent manner by SHHWE, whereas the level of interferon γ (IFN-γ), the atopy-related Th1 cytokine inhibiting the production of Th2 cytokines, was increased. Therefore, these results show that SHHWE has a potent anti-inhibitory effect on AD and is highly valuable for cosmetic development.

## Introduction

Atopic dermatitis (AD) is a chronic inflammatory disease associated with skin hypersensitivity caused due to environmental factors [1]. In AD patients, the skin barrier is significantly destroyed, and these patients have increased susceptibility to microbial colonization, infection, and allergic sensitization [2]. Eczema in AD has symptoms of delayed skin hypersensitivity, such as allergic contact dermatitis and hive (urticarial) lesions [3]. AD is a type II helper T lymphocytes (Th2)-allergic-mediated disease and is characterized by mast cell activation, peripheral eosinophilia, abnormal immunoglobulin E (IgE) production and induction of Th2 lymphocytes expressing interleukine (IL)-13 and IL-4 cytokines. There are many IL-5, IL-4 and IL-13 mRNA-expressing cells in acute skin lesions but few IL-12 or interferon γ (IFN-γ) mRNA-expressing cells [1, 4]. High counts of eosinophils are common in severe atopic diseases owing to IL-5 activation of stem cells. IL-5 is also a known product of Th2 cells in allergic diseases. It is established that the activation of Th2 cells induced by allergies is superior to that of type I helper T lymphocytes (Th1) cells in the skin of AD patients. The Th2 cytokines IL-5 and IL-4 take an role in AD by increasing IgE production and eosinophils [3]. The alleviating effect of red corn extract on AD in DNCB-induced mice [5] and the anti-atopic activity of ethanol extract from tuna heart [6] that produce decreased IFN-γ and enhanced proportion of allergen-specific T cells associated with increased IL-5 and IL-4 have been reported in peripheral blood smears of AD patients. The cause of atopy is not yet known, but genetic, immunological , and environmental factors are known to predispose patients to this disorder [7]. Moisturizers and systemic steroids and antihistamines for topical application are used for the therapy of AD, but some patients do not react well and have clinical limitations due to side effects, such as capillary dilatation, acne [8]. Thus, there is a pressing need to develop a natural product that can last for a long time minimizing the side effects and relieving the symptoms of atopic dermatitis. Seaweeds contain a large amount of minerals and a high content of polysaccharides among various marine organisms living in the ocean, and their importance as a natural resource with various physiological activities is gradually drawing attention [9]. *Sargassum horneri* is a brown alga belonging to Sargassaceae family found along the warm water coast of China. In addition to its use as food, it has also been utilized in Korean traditional medicine for centuries [10]. *Sargassum fulvellum* plays a picotal role in spawning and hideout for many marine animals by forming a sea forest but also causes many problems, such as damage to farms, emanation of odor, and landscape damage due to its accumulation on the coast [11]. *Sargassum horneri* also causes some problems since they are rarely consumed for food and most of them are discarded in Korea. However, it is abundant in vitamins, flavonoids, amino acids, polyphenols and polysaccharides, so it is worth studying. Therefore, various studies have been performed to increase the utilization of *S. Horneri* and their antioxidant [12], anti-inflammatory effect [10], and immune activity-enhancing effects [13]. To date, few studies on the physiological activity of the hot water extract of *S. horneri* have been recorded. Therefore, in this study, *S. horneri* hot water extract was applied to a DNCB-induced atopic mouse model, and atopy-related cytokine secretion in cell culture, total IgE secretion in serum, and visual evaluation were performed. In addition, the amount of cytokine secretion produced after treatment of normal mouse splenocytes with concanavalin A (Con A) and hot water extract was assessed. From these results, we attempted to reveal the potent anti-atopic activity and potential of *S. horneri* hot water extract as a functional natural resource.

## Materials and Methods

### Plant Materials and Extraction

Dried *S. horneri* was obtained from Wando-gun (Republic of Korea). Two kg of dried and crushed *S. horneri* were extracted for 4 h at 90–95°C using 64 L of purified water and filtered through filter paper (No. 4, Whatman International Ltd., UK). The filtrate was evaporated to 20% brix by using rotary evaporator (EYELA N-3010; Japan) at 50°C. The residue was lyophilized into powder (freeze dryer, FD8508, Il Shin Bio Base Co. Ltd., Korea) and stored at -20°C until use.

### Animals and Treatment

Five-week-old male BALB/c mice were purchased from Orient Bio Inc. (Republic of Korea a) and used after preliminary breeding in an animal room (50 ± 10% humidity, 20 ± 2°C temperature, and 12/12-h light–dark cycle) for a week. All experimental protocols for animal care were performed in accordance with the rules and regulations of the Animal Ethics Committee of Pukyong National University (Approval No. 201504).

### Induction of AD-Like Skin Lesions and Treatment

AD are induced as follows. After shaving the dorsal skin of 6-week-old male BALB/c mice, letted them unattended for 24 h to heal. To induce AD-like skin lesions, 200 μl of 1% DNCB (Sigma Chemical Co., USA) solution dissolved in an acetone and olive oil mixture (3:1) was used to sensitize the ears and dorsal skin regions at intervals of two days a week. A week after sensitizatoion, 200 μl of 0.3 % DNCB solution was evenly spread on the ears and dorsal skin once a day, and 30 mg/ml of hot water extracts (SHHWE) or 0.01% dexamethasone(Sigma Chemical Co.) 200 μl was topically applied to the same regions for 2 weeks at 12-h intervals with DNCB application. Mice were killed in the 3 week after DNCB sensitization.

### Splenocyte Culture

One day after the end of the treatment, five mice from each experimental group (normal, NC: negative control, PC: positive control, and SHHWE) were anesthetized by diethyl ether, and the samples were sterilized. Homogenize the spleen washed with RPMI medium (Medium 1640, Gibco, USA) with a tissue grinder and isolate the cells by filtering the homogeneous liquid with a cell strainer. The cell suspension was placed in RBC lysis buffer (Tris-buffered ammonium chloride; 0.87% (w/v) NH_4_Cl, pH 7.2) for 10 min for red blood cells (RBCs) lysis. The cells were seeded at 2 × 10^6^ cells/ml in 10% fetal bovine serum-RPMI medium and incubated in a 5% CO_2_ incubator (MCO-15 AC, Sanyo, Japan) at 37°C for 72 h.

### Cell Viability Assay

Splenocyte suspensions (2 × 10^6^ cells/ml) were cultured in 96-well plates 5% CO_2_ and at 37°C for 70 h. For the in vitro test, cells were cultured in SHHWE (0.1, 1, 10, 50, and 100 μg/ml) at 37°C for 70 h. After 70 h incubation, 5 mg/ml MTT (thiazol blue tetrazolium bromide, Sigma Chemical Co.) reagent was added to induce the formation of formazan crystals, followed by incubation for 2 h. To remove the medium, the plate was centrifuged at 2,000 rpm at 4°C for 10 min, and then replaced with dimethyl sulfoxide (DMSO) (Sigma Chemical Co.), and shaken for 20 min. The resulting color was assayed at 540 nm using a microplate reader (model 550; Bio-Rad, USA). Cell viability was calculated using the following equation:

Proliferation index (%) = Sample absorbance/Control absorbance×100

### Blood Collection

After collecting the spleen sample, approximately 1.0 ml of blood was collected from the aorta using a disposable syringe (Sungshim Medical Co., Ltd., Korea). For sera separation, blood was centrifuged at 10,000 rpm for 10 min at 4°C. The isolated sera were stored at -20°C until use.

### Serum IgE and Cytokine Measurement

Total IgE cytokine levels in sera and detection of IFN-γ, IL-5, and IL-4 in cultured splenocytes were performed using a mouse ELISA kit purchased from BD Biosciences (USA). It was measured and quantified in accordance with the protocol provided by BD Biosciences.

### Evaluation of Clinical Severity of Skin Dermatitis

The clinical severity of dermatitis on the dorsal skin was scored 0 days after the application of the test substance. The development of edema/excoriation, pruritus/dryness, erythema, erosion, and lichenification was scored as 0 (none), 2 (moderate), and 3 (severe) and summed up to give a score between 0 and 15 points [14]. A clinical severity score was defined by summing the individual scores for each group.

### Statistical Method

Statistical analysis for all experiments was performed using SAS software (Statistical Analytical System V8.2, SAS Institute Inc., USA) using the one-way ANOVA method. Significance was confirmed by Duncan's multiple comparison test at *p* < 0.05, and data are expressed as mean ± standard deviation.

## Results 

### Effect of SHHWE on Splenocyte Viability in DNCB-Induced BALB/c Mice

**Fig. 1:**
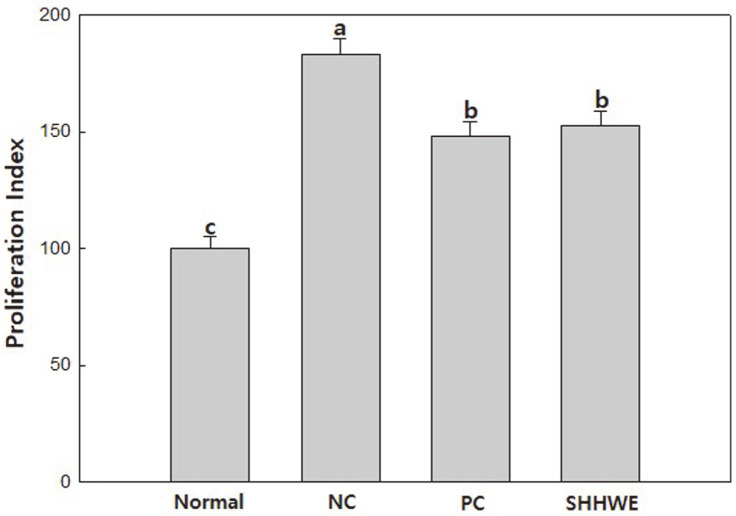
*Sargassum horneri* hot water extract (SHHWE) on the proliferation in splenocytes from atopic dermatitislike skin lesions mice. Normal, Negative control (NC: DNCB treated), Positive control (PC: DNCB and dexamethasone treated), SHHWE (DNCB and SHHWE treated). a-cMeans with different superscripts are significantly different (*p* < 0.05).

The MTT assay was performed by separating and culturing spleen cells after applying SHHWE to mice with AD induced by DNCB for 2 weeks. As a result (Fig. 1), the proliferation of splenocytes in the atopy-induced group treated with DNCB significantly increased, whereas the DNCB and SHHWE treatment showed a decrease to a level similar to that of the test group treated with DNCB and dexamethasone (DEXA), and the proliferative capacity was significantly lower at about 83% compared to the DNCB-alone treated group. Therefore, it was confirmed that SHHWE used in this study inhibited the proliferation of splenocytes stimulated by DNCB and showed no cytotoxicity.

### Effects of SHHWE on the IFN-γ, IL-5, and IL-4 Concentration in BALB/c Mice Splenocytes

**Fig. 2:**
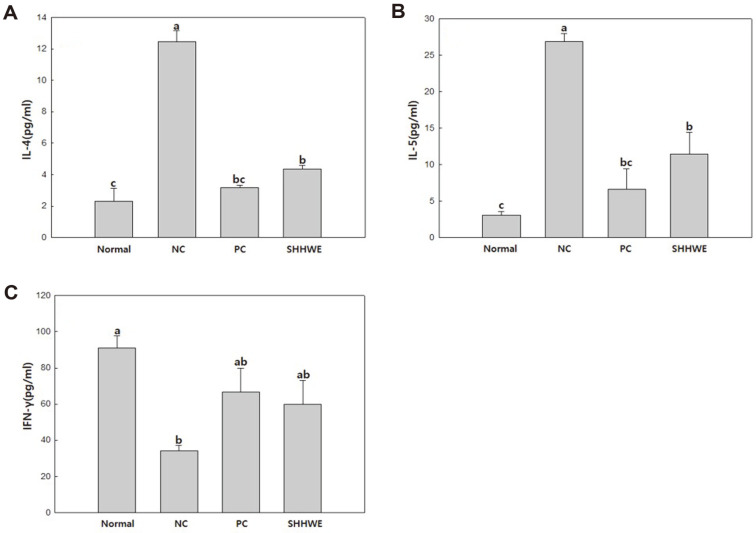
Effects of *Sargassum horneri* hot water extract (SHHWE) on the production of total IL-4 (A), IL-5 (B), IFN-γ (C) in mice splenocytes. Normal, Negative control (NC: DNCB treated), Positive control (PC: DNCB and dexamethasone treated), SHHWE (DNCB and SHHWE treated). ^a-c^Means with different superscripts are significantly different (*p* < 0.05).

When an external allergen invades the body, the number of cells expressing Th2 cytokines, such as IL-4 and IL-5, increases significantly, and B cells are stimulated to generate immunoglobulin E (IgE) due to the disruption of the balance between Th1 and Th2 cytokines. Among the Th2 cytokines, it is known that IL-4 and IL-5 are associated with the severity of AD [1,15]. Peripheral blood lymphocytes in AD patients secrete increased amounts of IL-4 cytokines and express abnormally high levels of IL-4 receptors [16]. In addition, in acute atopic lesions, IFN-γ, a Th1 cytokine, acts as a strong inhibitor of Th2 cell proliferation, IL-4 receptor expression in T cells and IgE synthesis. [17]. In this study (Figs. 2A and 2B), the levels of IL-4 and IL-5 tended to increase significantly in the DNCB-alone treated group. Moreover, the SHHWE group significantly reduced the level of each cytokine by about 35% and 42% compared to the DNCB group. In patients with acute AD, the production of IFN-γ, a Th1 cytokine, is reduced, while the production of Th2 cytokines, IL-4, is increased compared to normal people and resulted in an increase in IgE production [18]. The secretion of IFN-γ was decreased to about 66% compared to the normal group (Fig. 2C), but it was confirmed that similar level of difference existed compared to DEXA group. These observations suggested that AD can be suppressed by regulating the expression of each related cytokines of Th1 cells and Th2 cells in BALB/c mouse treated with SHHWE. In addition, the SHHWE group significantly reduced the level of each cytokine by about 35% and 42% as contrasted with the DNCB group, similar to the DEXA group.

### Effects of SHHWE on IFN-γ, IL-5, and IL-4 Production in Splenocytes Stimulated with Con A

**Fig. 3:**
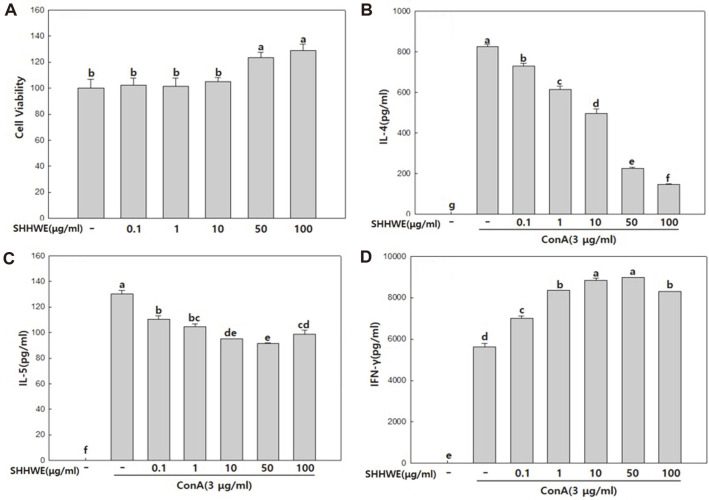
Effects of *Sargassum horneri* hot water extract (SHHWE) on the proliferation (A) and production of total IL-4 (B), IL-5 (C), IFN-γ (D) of mice splenocytes. ^a-g^Means with different superscripts are significantly different (*p* < 0.05).

To confirm the cytotoxicity of SHHWE, splenocytes of normal mice were separated, and SHHWE was treated at different concentrations (0.1, 1, 10, 50, and 100 μg/ml) and incubated for 70 h, after which the cell proliferation of splenocytes was measured. As a result (Fig. 3A), the survival rate at 50 and 100 μg/ml increased in a concentration-dependent manner, by approximately 123.5% and 129%, respectively, compared to control (100%). The spleen is in charge of immune defense against antigens in the blood, and there are various lymphocytes such as B cells that cause somatic immune responses, T cells related to cellular immune responses, and macrophages. The proliferation of spleen cells is important in immune response because stimulating immune active substances through the inflow of antigens induces the proliferation and differentiation of lymphocytes in the spleen [19]. Therefore, it is thought that SHHWE used in this study acts as a mitogenic factor on splenocytes to increase their proliferation and activating immune cells by not being cytotoxic. The most important pathological mechanism of atopy is the adverse reaction of the immune system characterized by allergic inflammation with a predominant Th2 cell environment [20]. In particular, the immune response of Th2 lymphocytes mediated by IL-13, IL-4, and IL-5 plays a pivotal role in atopic lesions [21]. Immunological feature of AD is known as Th1/ Th2 imbalance. IL-13 and IL-4 promote IgE synthesis by activating B cells, and the connection between increased in IgE levels and Th2 cytokines has been reported in previous studies [3, 22, 23]. IL-5 increases eosinophil hyperplasia and infiltration into lesions and regulates eosinophil activity [24]. Eosinophilic hyperplasia of tissues is characterized by atopic disease and is considered a major effective cell in the late immune response of patients with AD repeatedly exposed to allergic antigens [3]. Th1 cells generate IFN-γ to promote cell-mediated immunity and to control intracellular pathogens [25]. IFN-γ, a Th1 cytokine, strongly inhibits the expression of IL-4 in T cells and suppresses the synthesis of IgE, which is stimulated by IL-4. SHHWE mice were treated with different concentrations (0.1, 1, 10, 50, and 100 μg/ml) to observe the secretion of IL-4 and IL-5, cytokines of Th1 and Th2, IFN-γ, Con A, and T-cell mitogenic factor-induced proliferation in the cell culture of splenocytes of normal mice As a result (Fig. 3), IL-4 secretion by spleen cells increased significantly when Con A was used alone but decreased in a concentration-dependent manner with SHHWE treatment, showing significant inhibitory effects up to 27.3% and 17.6% at 50 and 100 μg/mL concentrations, respectively (Fig. 3B). In the case of IL-5, it was not in a concentration-dependent manner; however, it was confirmed that secretion decreased after treatment with SHHWE to 70.1% and 75.8% at concentrations of 50 and 100 μg/mL, respectively (Fig. 3C). In contrast, the secretion of IFN-γ, a Th1 cytokine that inhibits IL-4 and IgE, increased by 56.8%, 59.6%, and 47.3%, respectively, in the 10, 50, and 100 μg/ml treatment of SHHWE compared to Con A treatment alone. Contrary to IL-5 secretion, a slightly decreased level at 100 μg/mL was confirmed, suggesting that the opposite result was obtained at the same concentration owing to the correlation between Th1 and Th2 cell immune regulation (Fig. 3D). Therefore, SHHWE is believed to suppress symptoms of AD by regulating the expression of cytokines secreted from Th1 and Th2 cells.

### Effects of SHHWE on DNCB-Induced IgE Levels in BALB/c Mice Sera

**Fig. 4:**
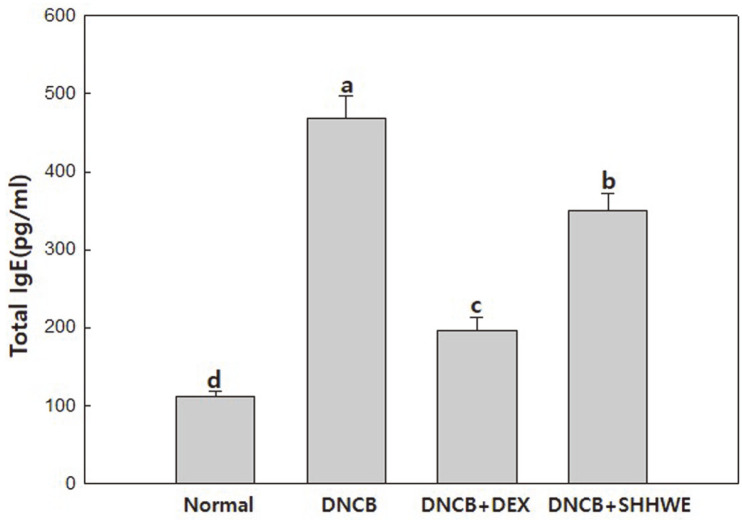
Effects of *Sargassum horneri* hot water extract (SHHWE) on the production of total IgE in mice sera. Normal, Negative control (NC: DNCB treated), Positive control (PC: DNCB and dexamethasone treated), SHHWE (DNCB and SHHWE treated). Values are mean ± SD. ^a-d^Means with different superscripts are significantly different (*p* < 0.05).

Various systemic and skin immune abnormalities related to atopic dermatitis, such as increased sensitivity to allergens and serum IgE, increased T cells expressing skin lymphocyte antigens, and increased expression of Th2 cytokines in acute lesions have been identified to date. Additionally, in patients with AD, the expression of FceRI increased in the epidermis of Langerhans and dendritic cells, and the expression of antimicrobial peptides decreased [2]. Th2 cytokines are overproduced due to the invasion of external allergens, which stimulate B cells to produce immunoglobulin E (IgE). Excessively increased IgE migrates to mast cells in the skin lesion, cross-linked to the FceRI receptor on the cell surface. When repeatedly exposed to the same allergen, the specific IgE attached to the FceRI receptor binds to the allergen, thereby sensitizing mast cells. At this time, as mast cells receive the same stimulus and with broken cell surface, degranulation of inflammatory factors, such as heparin and histamine, occurs. Excessive secretion of these degranulation substances induces and exacerbates AD accompanied by allergic symptoms, such asfa severe itching, vasodilation, red spots, and edema [1]. Therefore, overexpression of IgE in the blood is a typical feature of atopic lesions, and the synthesis of IgE produced in B cells is promoted by IL-4 and Th2 cells [21]. However, it is known that IgE promoted by IL-4, a Th2 cytokine, is inhibited by Th1 cell cytokines, such as IFN-γ. In this study, AD was induced by DNCB, and the IgE content in BALB/c mouse serum was quantified by ELISA after applying SHHWE. As shown in Fig. 4, the total IgE content was 468.79 ± 27.88 pg/ml, showing the highest value in the DNCB-alone treated group. In the SHHWE group, the content was 350.61 ± 21.92 pg/ml, and it was confirmed that the total IgE in the blood significantly decreased by the SHHWE treatment compared to the total IgE of the DNCB alone treatment. Associated with the results of inhibiting the production of the Th2 cytokines IL-5 and IL-4, the production of IgE is believed to be suppressed by Th1 cytokines and increased by Th2 cytokines. Therefore, it is assumed that the application of SHHWE may alleviate the symptoms of atopic dermatitis.

### Effect of SHHWE on DNCB-Induced Clinical Severity

**Fig. 6:**
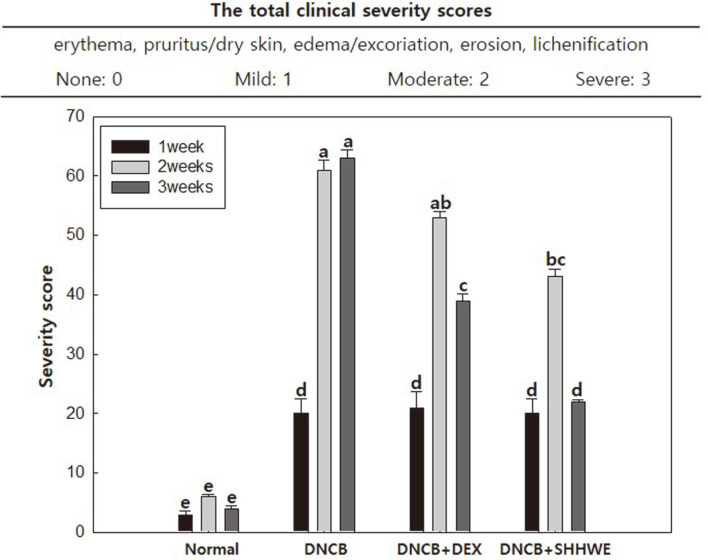
Effects of *Sargassum horneri* hot water extract (SHHWE) on clinical score of DNCB-applied BALB/c mice (*n* = 5). Normal, Negative control (NC: DNCB treated), Positive control (PC: DNCB and dexamethasone treated), SHHWE (DNCB and SHHWE treated). Values are mean ± SD. ^a-d^Means with different superscripts are significantly different (*p* < 0.05).

To confirm the degree of AD recovery in SHHWE, DNCB-induced mice were divided into three groups: normal control, treated with DNCB alone, and treated with DEXA and SHHWE. The backs of the mice were then observed to check the degree of recovery from atopic dermatitis. During the first week of DNCB treated alone, there were no noticeable differences in smudges, erythema, skin dryness, edema, and bleeding in the applied area except for the control. However, the DNCB and DEXA treatment recovered to some extent compared to the DNCB treatment alone, and it was confirmed that the dermatitis of the affected area recovered to a nearly normal skin during the 3rd week after 2 weeks of continuous application. In addition, according to the clinical visual evaluation for atopic dermatitis [14], the total score for evaluating five items, erythema, itching and dry skin, edema, squabbling, and lichenification, is shown (Fig. 6). The severity score graph also showed a similar pattern to the visual estimate. Erythema and lichenification were observed when applied for 1 week, and there was no meanigful difference in comparison the DNCB treatment alone. As a result of continuous application of SHHWE for 2 weeks, it was confirmed that the score considerably decreased in comparison the DNCB-treated group. These results were similar to those reported on the alleviation of AD in a study on the anti-atopic effects of *Sargassum fulvellum* water extract [27]. Therefore, SHHWE is considered to be effective in improving dry skin by regulating the inflammatory response through the cellular responses of Th1 and Th2.

## Discussion

AD is a common, severely pruritic, chronic or chronically relapsing, eczematous skin disease mostly associated with mast cell activation, hyperimmunoglobulin E syndrome, and eosinophilia. Oral antihistamines, softeners, and topical steroids are used as primary treatments for atopic dermatitis; however, the side effects of long-term use of topical steroids have caused concern worldwide. Topical corticosteroids commonly used for AD cause a variety of skin side effects in local applications, such as capillary dilatation, skin atrophy, polycythemia, mouth-peripheral dermatitis, fungal/bacterial/virus infections, and atrophic skin progeny [27]. While research on the cause of AD is underway and new treatments are being developed, anti-atopic drugs currently have side effects, limiting their clinical applications [17]. Although many drugs with various natural materials have recently been used, studies proving their efficacy in treating AD are insufficient. The AD-like skin lesions in mice are similar to those in humans. In this study, we investigated whether SHHWE attenuated the clinical severity of DNCB-induced AD-like skin lesions. Signs and symptoms include erythema and bleeding in various grades, followed by edema, deep abrasions, surface erosion, and dry skin [28]. Mouse models generally show signs similar to those of human immunological abnormalities, such as IgE overexpression in the serum. The results of this study demonstrated that continuous local application of SHHWE to DNCB-sensitive BALB/c mice significantly inhibited the symptoms of AD-like skin lesions. Therefore, total IgE levels in the serum and Th1 and Th2 cytokine secretion were measured. Visual evaluation and skin clinical severity scoring were also performed. Although the etiology of AD is thought to be caused by the complex multifactorial interaction of environmental and genetic factors, it has not been clasrified. To date, two hypotheses have been proposed: one is an immunomodulatory disorder causing inflammation and IgE-mediated sensitization, wherein Th2 cells are dominant, and the other is an essential defect in the skin barrier function as the main cause. In the latter case, even in the stage unrelated to the disease, skin hypersensitivity appears on the skin without lesions due to the genetic predisposition of skin barrier. This AD is characterized by IgE-mediated sensitization, impaired skin barrier and skin inflammationand to allergens in food and the environment. Histologically, acute eczematous lesions show cavernosis, hyperkeratosis, and parakeratosis, while chronic lesions are characterized by vascular infiltration and acanthosis [29]. The response of Th1 and Th2 cells, characterized by Th2 cells and predominant allergic inflammation, is key to atopic pathology. In particular, the Th2 immune response mediated by IL-4, IL-5, and IL-13 plays an important role. IL-5 plays a role in eosinophil infiltration, and IL-4 and IL-13 activate B cells to promote IgE synthesis. IL-4 expression plays a major role in the elevation of IgE levels. The results of cytokine analysis in the mouse model suggest that SHHWE reduced the production of total IgE in the serum by inhibiting the production of Th2 cytokines, such as IL-4 and IL-5, associated with an increase in IgE levels (Figs. 2A, 2B, and 4). IFN-γ was analyzed as a biomarker to evaluate the therapeutic potential of SHHWE. IFN-γ is a major cytokine produced by Th1 cells acting as an immune regulator and in patients with atopic dermatitis, the level of IFN-γ is generally reduced [30]. Based on the results of this study (Fig. 2C), SHHWE is thought to inhibit skin lesions in the DNCB-induced AD mouse model by increasing the production of IFN-γ. Lectins are proteins that bind to sugar residues on the surface of various types of cells and have biological properties, such as aggregation of bacterial, plant, or mammalian cells, stimulation of T lymphocyte mitosis, and induction of cytotoxicity, in lymphocytes and macrophages. The lectin-induced cytotoxic action of T lymphocytes is due to their ability to affect immunologically unrelated target cells, link effector and target cells, and activate T lymphocytes to kill target effector cells [31]. Con A, a representative lectin, is extensively used to activate T cells. The exact mechanism of action is not yet known, but Con A is most likely to activate T cells through indirect crosslinking of the T cell receptors [28]. T cells regulate immune functions by secreting various cytokines. Among them, Th1-type T cells promote cellular immunity by producing IL-2, tumor necrosis factor (TNF), and IFN-γ, and Th2 cells producing cytokines, such as IL-4, IL-5, IL-6, and IL-10, by promoting humoral immunity through antibody production. In this study, SHHWE restrained the expression of IL-4 and IL-5 in the splenocytes of normal mice activated with Con A and increased IFN-γ production by Th1 cells. These results (Fig. 3) suggest that SHHWE is not cytotoxic and has an atopic inhibitory effect by inhibiting T cell activation. Several studies have reported that Th1 and Th2 lymphocytes exhibit antagonistic action, and when the function of Th1 lymphocytes is increased, the function of Th2 lymphocytes is suppressed. IFN-γ produced by Th1 lymphocytes not only has antiviral activity but also increases class II MHC production and decreases CD23 expression. It also inhibits IgE production by antagonizing the action of IL-4. In this study, SHHWE suppressed the expression of IL-4 and IL-5 in the splenocytes of normal mice activated with Con A and increased IFN-γ production by Th1 cells. These results (Fig. 3) also suggest that SHHWE is not cytotoxic and has inhibitory activity on AD by inhibiting T cell activation. Consequently, topical application of SHHWE reduced serum IgE levels and inhibited the development of AD-like skin lesions. The inhibitory effect of SHHWE on skin lesion development appears to be due to its modulating effects on cytokines such as IFN-γ, IL-4, and IL-5. Visual evaluation confirmed that the symptoms of AD were remarkably alleviated when SHHWE was applied continuously for 2 weeks, and the value significantly reduced in the skin clinical severity score compared to the DNCB treated alone. These results indicate that SHHWE has excellent efficacy in improving AD by regulating the generation and suppressing the overexpression of IgE and activity of Th2 and Th1 cytokines.

## Figures and Tables

**Fig. 5:**
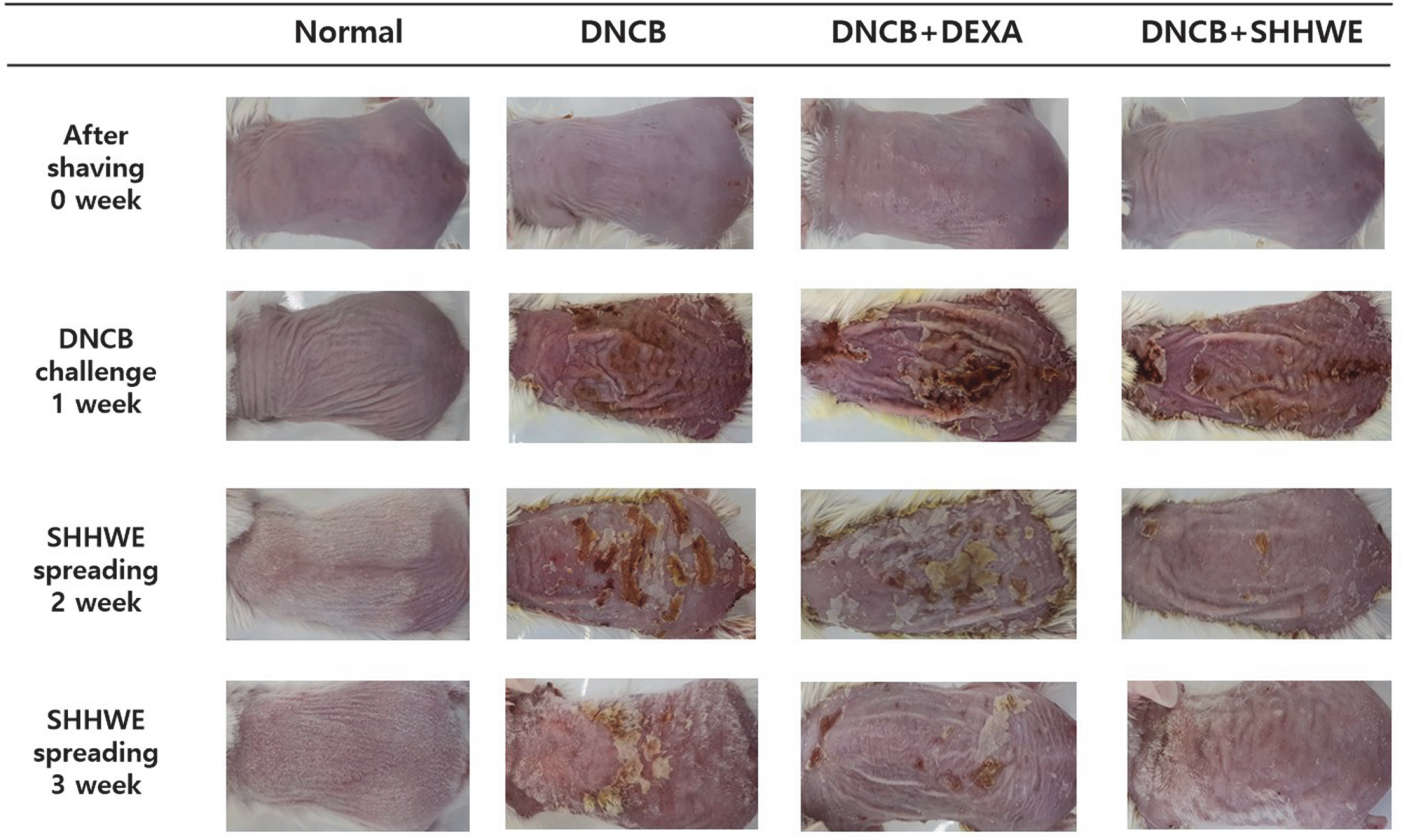
Effects of *Sargassum horneri* hot water extract (SHHWE) on clinical skin features with photograph taken every week of DNCB-applied BALB/c mice (*n* = 5). Normal, Negative control (NC: DNCB treated), Positive control (PC: DNCB and dexamethasone treated), SHHWE (DNCB and SHHWE treated).
